# Prognostic and clinicopathological significance of fatty acid synthase in breast cancer: A systematic review and meta-analysis

**DOI:** 10.3389/fonc.2023.1153076

**Published:** 2023-04-12

**Authors:** Binyan Liu, Qi Peng, Ya-Wen Wang, Jianhao Qiu, Jiang Zhu, Rong Ma

**Affiliations:** ^1^ Department of Breast Surgery, Qilu Hospital of Shandong University, Jinan, Shandong, China; ^2^ Department of Thoracic Surgery, Qilu Hospital of Shandong University, Jinan, Shandong, China

**Keywords:** fatty acid synthase (FASN), breast cancer, disease-free survival (DFS), relapse-free survival (RFS), overall survival (OS), clinicopathology, meta-analysis

## Abstract

**Background:**

Aberrant expression of fatty acid synthase (FASN) was demonstrated in various tumors including breast cancer. A meta-analysis was conducted to investigate the role of FASN in breast cancer development and its potential prognostic significance.

**Methods:**

The Web of Science, PubMed, Embase, and Cochrane Library databases were searched to identify studies that evaluated the relationship between FASN expression and overall survival (OS), relapse-free survival (RFS), and disease-free survival (DFS) of breast cancer patients. To analyze the clinicopathological and prognostic values of FASN expression in breast cancer, pooled hazard ratios (HRs), odds ratios (ORs), and 95% confidence intervals (CIs) were clustered based on random-effects models. To confirm whether the findings were stable and impartial, a sensitivity analysis was performed, and publication bias was estimated. Data were analyzed using Engauge Digitizer version 5.4 and Stata version 15.0.

**Results:**

Five studies involving 855 participants were included. Patients with higher FASN expression did not have a shorter survival period compared to those with lower FASN expression (summary HR: OS, 0.73 [95% CI, 0.41-1.32; P=0.300]; DFS/RFS, 1.65 [95% CI, 0.61-4.43; P=0.323]). However, increased FASN expression was correlated with large tumor size (OR, 2.04; 95% CI, 1.04-4.00; P=0.038), higher human epidermal growth factor receptor 2 (HER2) positivity (OR, 1.53; 95% CI, 1.05-2.23; P=0.028). No significant associations were observed between FASN expression and histological grade (OR, 0.92; 95% CI, 0.41-2.04; P=0.832), Tumor Node Metastasis (TNM) stage (OR, 1.11; 95% CI, 0.49-2.53; P=0.795), nodal metastasis (OR, 1.42; 95% CI, 0.84-2.38; P=0.183), Ki-67 labelling index (OR, 0.64; 95% CI, 0.15-2.63; P=0.533), estrogen receptor (ER) status (OR, 0.90; 95% CI, 0.61-1.32; P=0.586), or progesterone receptor (PR) status (OR, 0.67; 95% CI, 0.29-1.56; P=0.354).

**Conclusion:**

FASN is associated with HER2 expression and may contribute to tumor growth, but it has no significant impact on the overall prognosis of breast cancer.

## 1 Introduction

Breast cancer is the most common malignant tumor in women globally (1) and has four histological subtypes: triple-negative, human epidermal growth factor receptor 2 (HER2)-overexpress, luminal A, and luminal B (2–4). Significant prognostic differences exist among these four subtypes (5, 6). The prognosis for luminal breast cancer is favorable because it can be treated with long-term endocrine therapy and chemotherapy. Targeted medications can be used to treat HER2-overexpress breast cancer and improve its prognosis (7–9); however, triple-negative breast cancer is associated with a worse prognosis and shorter survival period (10, 11), partly because of the lack of effective drugs (12). Recently, researchers have focused on identifying better treatment targets and biological indicators to predict breast cancer.

Due to the wide use of mammography and breast ultrasonography, more early-stage breast cancer cases are being discovered, and the 5-year overall survival (OS), relapse-free survival (RFS), and disease-free survival (DFS) rates of these patients are greatly improved (13). However, the predictive indicators for accurate prognostic assessment before treatment are still lacking.

Recent studies show that fatty acid metabolism has been linked to the clinical prognosis of various malignancies and implicated in their aetiology (14–16). Cancer cells cannot obtain sufficient energy solely through glycolysis (17); therefore, they often synthesis endogenous fatty acids for energy supply (18). Fatty acid synthase (FASN) is the primary enzyme for *de novo* endogenous synthesis of fatty acids. The fatty acids synthesized by FASN not only provide energy, but also participate in signal transduction in the cellular membrane of breast cancer cells (19, 20). FASN promotes the formation of the lipid raft phospholipid HER2 transduction complex on cell membranes by catalyzing fatty acid synthesis, which is subsequently involved in activating the PI3K (phosphoinositide 3-kinase)/AKT/mTOR (mammalian target of rapamycin) pathways (19, 20). FASN overexpression was found in many types of tumor cells including breast cancer (21–23). Whether FASN expression had impact on the prognosis in patients with breast cancer is elusive. There is a study showing that increased FASN expression is correlated with shorter survival (24); however, such a correlation has not been demonstrated by other study (25). It is also suggested that higher FASN expression may be linked to a worse prognosis for some specific subtypes of breast cancer (26). To better comprehend the function of FASN in the development and prognosis on breast cancers, a meta-analysis was conducted based on the accessible literature.

## 2 Methods

### 2.1 Protocol and ethics statement

The results of this study have been reported in accordance with the guidelines and statements of the systematic review and meta-analysis of preferred reporting projects and meta-analyses of observational epidemiological studies (27, 28). Additionally, it was registered on the INPLASY website (https://inplasy.com/inplas-2022-12-0020; registration number: INPLASY2022120020). All data used for this meta-analysis were obtained from published studies; therefore, the requirements for ethical approval and patient consent were waived.

### 2.2 Search strategy

Using the Embase, Web of Science, PubMed, and Cochrane Library databases, a thorough search of the literature was conducted to find papers that evaluated the relationship between FASN and OS or DFS/RFS of breast cancer patients and were published before September 1, 2022. Only articles published in English were included in this search. “Breast cancer,” “fatty acid synthase,” “survival,” “mortality,” and “prognosis” were used as keywords during the investigation, which included free-text words and Medical Subject Headings/EMTREE phrases. The review publications and reference lists of all pertinent studies were manually searched for additional records not found during the database search. **Table S1** describes the search strategies used for each database.

### 2.3 Inclusion and exclusion criteria

The inclusion criteria were as follows: (I) involved patients diagnosed with breast cancer histopathologically; (II) hazard ratios (HRs) and corresponding 95% confidence intervals (CIs) for FASN and survival outcomes were reported, or other data for the reconstruction of survival data, such as Kaplan–Meier curves; (III) FASN expression was determined by immunohistochemistry.

The criteria for exclusion were as follows: (I) ineligible article types including reviews, meta-analyses, case reports, conference abstracts, letters and comments; (II) animal studies or basic research; (III) studies without sufficient data for analyses; (IV) studies from the same center with patients overlap; (V) data from public database.

### 2.4 Data extraction and quality assessment

Two researchers independently collected the following pertinent information from the available studies: first author’s last name; study location; study type; year of publication; study period; treatment; follow-up period; sample size; detection method; reported outcome; and HRs and 95% CIs. The Newcastle-Ottawa Quality Assessment Scale was used to assess the quality of evidence of each included study (29). Using a scale of 0 to 9, items with a score of more than 6 were considered high-quality. A detailed quality assessment of the included studies is presented in **Table S2**.

### 2.5 Statistical analysis

The predictive value of FASN expression in breast cancer was assessed using HRs and 95% CIs. The HRs and 95% CIs were retrieved directly if they were reported; otherwise, the HRs and 95% CI were derived from the available data using Engauge Digitizer version 5.4 (30).

In order to reflect the potential heterogeneity among the included research and minimize potential bias, a random-effects model was employed to pool the results (31). I^2^ statistics and Cochran’s test were used to assess the study heterogeneity. I^2^ > 50% indicated considerable heterogeneity. The consistency of the findings was evaluated using a sensitivity analysis. Begg’s regression and Egger’s linear regression were used to determine publication bias, and a visual assessment of funnel plot symmetry was performed (32, 33). Stata version 15.0 was used to conduct all statistical analyses (Stata Corporation, College Station, TX, USA).

## 3 Results

### 3.1 Literature search and study selection

**Figure 1:**
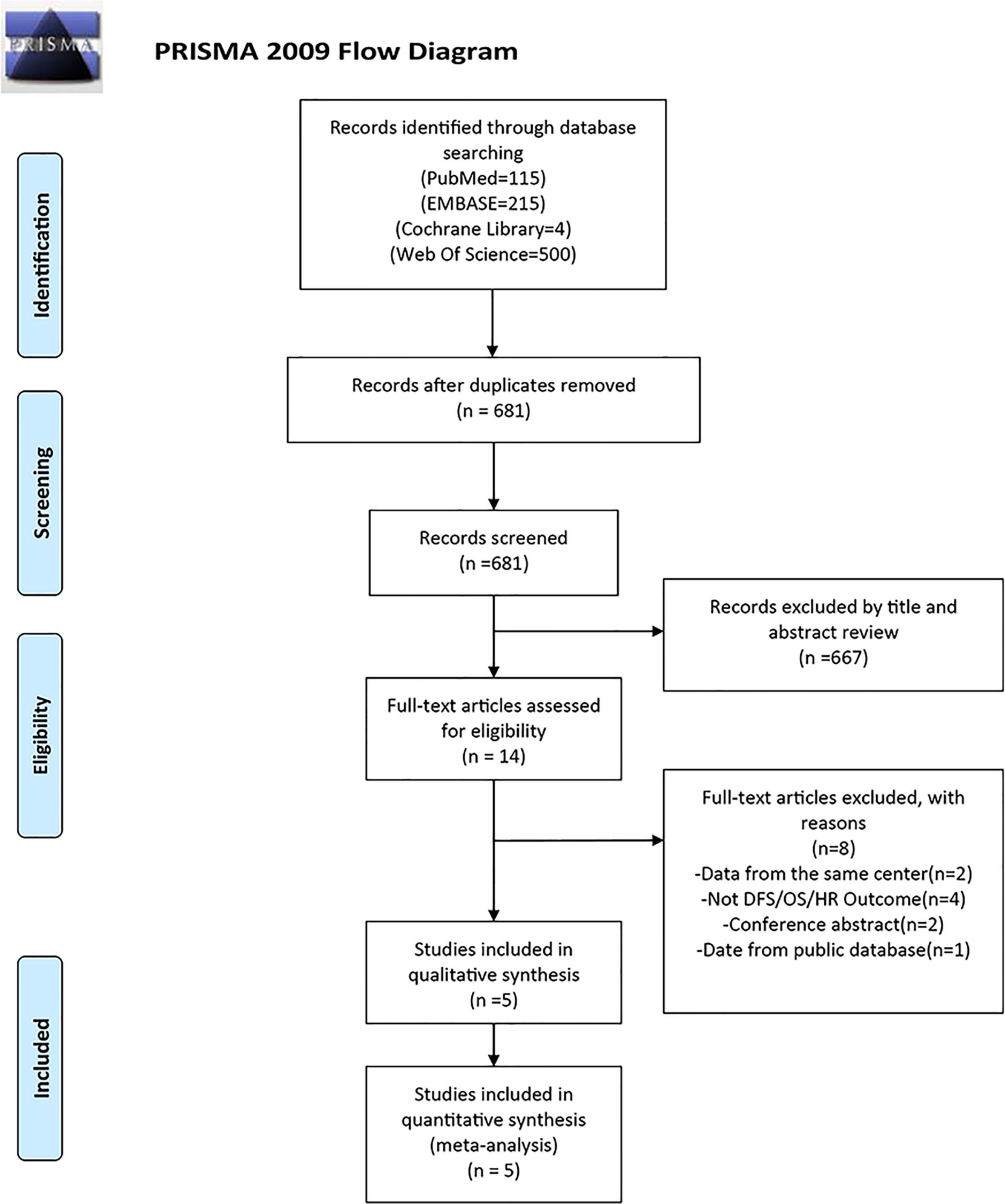
PRISMA flow diagram of literature retrieval. PRISMA, Preferred Reporting Items for Systematic Reviews and Meta-Analyses.

**Figure 1** displays a flowchart of the study selection procedure. A total of 834 possible studies were found; of these, 115 were in PubMed, 215 were in Embase, 500 were in the Web of Science, and 4 were in the Cochrane Library databases. After removing the duplicates, 681 studies were retained. Only 14 of these studies investigated the connection between FASN and the prognosis for breast cancer. Three studies were conducted at the same institution; therefore, only one was included. Four studies did not examine DFS, OS, or HRs, and two were conference abstracts. One study’s data was from the public database; therefore, they were excluded. Ultimately, the meta-analysis included five studies with 855 individuals (25, 34–37).

### 3.2 Study characteristics

**Table 1** lists the clinical features of the studies included in this meta-analysis. All studies were performed retrospectively between 1983 and 2018. These studies were performed in five countries (one in China, one in South Korea, one in Spain, one in Japan, and one in Italy). The tumor stage at diagnosis and characteristics of the cancer varied between studies. All studies used immunohistochemistry to distinguish the level of FASN expression. Two studies did not provide clinical information regarding the tumors or treatment. Three studies reported that the patients received only surgical treatment. One studies did not report age at enrollment; the mean age at enrollment reported by the remaining studies ranged from 50.5 to 68 years. The number of participants ranged from 61 to 476. The follow-up intervals for two investigations were 10 and 15 years, respectively; however, the other studies did not specify the follow-up period. The link between FASN expression and the clinicopathological characteristics of breast cancer was examined by five studies.

**Table 1:** Baseline characteristics of included studies.

Study (year)	Country	Study Period	Study type	Age	TNM stage	Follow-up Period	Treatment	Sample Size	Detection method	Outcome reported
Alo, P. L. et al., 1996 (34)	Italy	1983-1987	retrospective	56.7(37-87)	I	120	Surgery	110	immunohistochemistry	DFS
Hong, Y. et al., 2016 (35)	China	NR	retrospective	NR	I-IV	NR	NR	108	immunohistochemistry	OS
Yoshikawa, K. et al., 2022 (36)	Japan	2006-2018	retrospective	68(31-93)	I-IV	173	Surgery	61	Immunohistochemistry	RFS OS
Giró-Perafita, A. et al., 2017 (25)	Spain	1990-2012	retrospective	58.1(41.8-74.4)	I-III	NR	NR	100	immunohistochemistry	DFS OS
Kim, S. et al., 2015 (37)	Korea	2002-2006	retrospective	50.5(40.2-60.8)	I-III	NR	Surgery	476	immunohistochemistry	DFS OS

OS, overall survival; DFS, Disease free survival; TNM, Tumor Node Metastasis; NR, not reported.

### 3.3 FASN expression and the DFS/RFS of breast cancer patients

**Figure 2:**
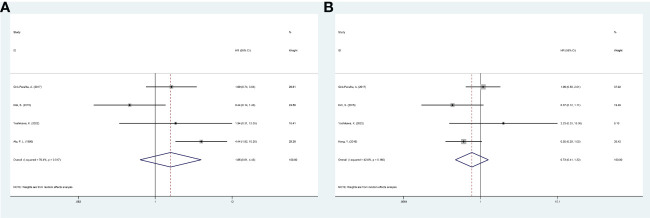
Meta-analysis of fatty acid synthase expression level and prognosis of breast cancer patients. **(A)** Forest plot of associations between fatty acid synthase expression level and DFS/RFS of patients. **(B)** Forest plot of associations between fatty acid synthase expression level and OS. DFS, disease-free survival; RFS, relapse-free survival; OS, overall survival.

Four studies reported a correlation between FASN expression and the DFS/RFS of breast cancer patients. The pooled analysis indicated, with significant heterogeneity (I^2^ = 70.4%; P = 0.017), that increased FASN expression was not associated with the poor DFS/RFS of breast cancer patients (HR, 1.65; 95% CI, 0.61-4.43; P = 0.323) (**Figure 2**). Based on nationality, the initial inclusion period, and median age, a subgroup analysis was conducted. High FASN expression was associated with the worse DFS/RFS in Europe (**Table 2**).

**Table 2:** Subgroup analyses of OS, DFS/RFS in breast cance.

Variable	No. of studies	No. of patients	Effects model	HR (95% CI)	*P*	Heterogeneity	
						I^2^	*P*
OS							
All	4	745	Random	0.73 (0.41-1.32)	0.300	42%	0.16
Country							
Asia	3	645	Random	0.57 (0.29-1.12)	0.104	21.8%	0.279
Europe	1	100	Random	1.09 (0.59-2.01)	0.782	–	–
Initial inclusion period							
Before year 2000	1	100	Random	1.09 (0.59-2.01)	0.782	–	–
After year 2000	2	537	Random	0.76 (0.14-4.26)	0.757	60.8%	0.110
NR	1	108	Random	0.55 (0.29-1.05)	0.069	–	–
Median Age							
<60	2	576	Random	0.70 (0.25-1.98)	0.505	64.8%	0.092
>60	1	61	Random	2.23 (0.33-15.06)	0.411	–	–
NR	1	108	Random	0.55 (0.29-1.05)	0.069	–	–
DFS/RFS							
All	4	747	Random	1.65 (0.61-4.43)	0.323	70.4%	0.017
Country							
Asia	2	537	Random	0.78 (0.19-3.20)	0.725	44.9%	0.178
Europe	2	210	Random	2.73 (1.06-7.03)	0.038	61.6%	0.107
Initial inclusion period							
Before year 2000	2	210	Random	2.73 (1.06-7.03)	0.038	61.6%	0.107
After year 2000	2	537	Random	0.78 (0.19-3.20)	0.725	44.9%	0.178
Median Age							
<60	3	686	Random	1.57 (0.47-5.27)	0.461	80.3%	0.006
>60	1	61	Random	1.94 (0.31-12.05)	0.477	–	–

HR, Hazard Ratio; OS, overall survival; DFS, disease-free survival; RFS, recurrence-free survival; NR, not reported.

### 3.4 FASN expression and the OS of breast cancer patients

Four studies investigated the link between FASN expression and the OS of breast cancer patients. The pooled analysis indicated, with heterogeneity (I^2^ = 42.0%; P = 0.160), that increased FASN expression was not linked to poor OS (HR, 0.73; 95% CI, 0.41-1.32; P = 0.300) (**Figure 2**). A random-effects model was used to combine the results. Based on nationality, the initial inclusion period, and median age, a subgroup analysis was conducted; high FASN expression was not correlated with the worse OS of the subgroups (**Table 2**).

### 3.5 Correlation between FASN and clinicopathological characteristics of breast cancer

**Figure 3:**
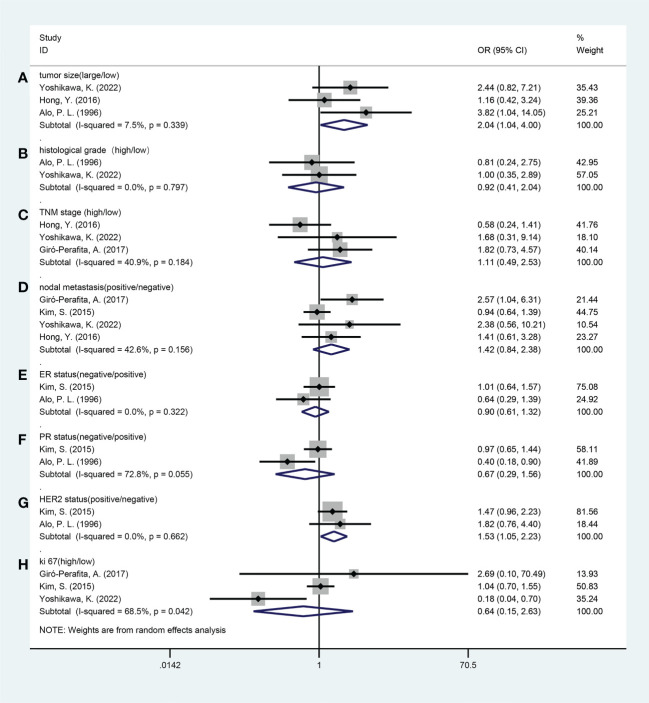
Forest plots of the relationship between fatty acid synthase expression level and clinicopathological features. **(A)** Tumor size (large vs. small); **(B)** Histological grade (high vs. low); **(C)** TNM stage (high vs. low); **(D)** Nodal metastasis (yes vs. no); **(E)** ER status (negative vs. positive); **(F)** PR status (negative vs. positive); **(G)** HER2 status (positive *vs.* negative); **(H)** Ki-67 (high vs. low). ER, estrogen receptor; PR, progesterone receptor; HER2, human epidermal growth factor receptor 2; OR, odds ratio; CI, confidence interval.

Five studies reported a relationship between FASN expression and the clinicopathological characteristics of breast cancer patients, including tumor size (large vs. small), histological grade (high vs. low), Tumor Node Metastasis (TNM) stage (high vs. low), lymph node metastasis (yes vs. no), Ki-67 labelling index (high vs. low), estrogen receptor (ER) status (negative vs. positive), progesterone receptor (PR) status (negative vs. positive), and HER2 status (positive vs. negative). The grouping of the above clinicopathological indicators by different studies is detailed in **Table S3**. The results of the pooled analysis demonstrated that increased FASN expression was associated with large tumor size (odds ratio [OR], 2.04; 95% CI, 1.04-4.00; P = 0.038), HER2 positivity (OR, 1.53; 95% CI, 1.05-2.23; P = 0.028). However, no significant associations were observed between FASN expression and the histological grade (OR, 0.92; 95% CI, 0.41-2.04; P=0.832), TNM stage (OR, 1.11; 95% CI, 0.49-2.53; P = 0.795), lymph node metastasis (OR, 1.42; 95% CI, 0.84-2.38; P = 0.183), Ki-67 labelling index (OR, 0.64; 95% CI, 0.15-2.63; P = 0.533), estrogen receptor (ER) status (OR, 0.90; 95% CI, 0.61-1.32; P = 0.586), or progesterone receptor (PR) status (OR, 0.67; 95% CI, 0.29-1.56; P = 0.354) (**Table 3**; **Figure 3**).

**Table 3:** Correlations of FASN and clinicopathological characteristics in patients with breast cancer.

Characteristics	No. of studies	No. of patients	Effects model	OR (95% CI)	*P*	Heterogeneity	
						I^2^, %	*P*
Tumor size (large vs. small)	3	279	Random	2.04 (1.041-4.00)	0.038	7.5	0.339
Histological grade (high vs. low)	2	171	Random	0.92 (0.41-2.04)	0.832	0	0.797
TNM stage (large vs. low)	3	255	Random	1.11 (0.49-2.53)	0.795	40.9	0.184
Lymph node metastasis (yes vs. no)	4	976	Random	1.42 (0.84-2.38)	0.19	42.6	0.156
Ki-67 (high vs. low)	3	835	Random	0.64 (0.15-2.63)	0.533	68.5	0.042
ER status (negative vs. positive)	2	850	Random	0.90 (0.61-1.32)	0.586	0	0.322
PR status (negative vs. positive)	2	850	Random	0.67 (0.29-1.56)	0.354	72.8	0.055
HER2 status (positive *vs.* negative)	2	850	Random	1.53 (1.05-2.23)	0.028	0	0.662

FASN, fatty acid synthase; TNM, Tumor Node Metastasis; ER, estrogen receptor; PR, progesterone receptor; HER2, human epidermal growth factor receptor 2; OR, odds ratio; CI, confidence interval.

### 3.6 Sensitivity analyses and publication bias

**Figure 4:**
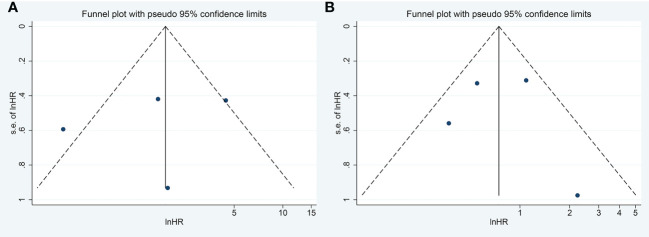
Funnel Plots to detect publication bias. **(A)** Funnel Plots to detect publication bias for meta-analysis of FASN expression level and DFS/RFS. **(B)** Funnel Plots to detect publication bias for meta-analysis of FASN expression level and OS. FASN, fatty acid synthase; DFS, disease-free survival; RFS, relapse-free survival; OS, overall survival.

**Figure 5:**
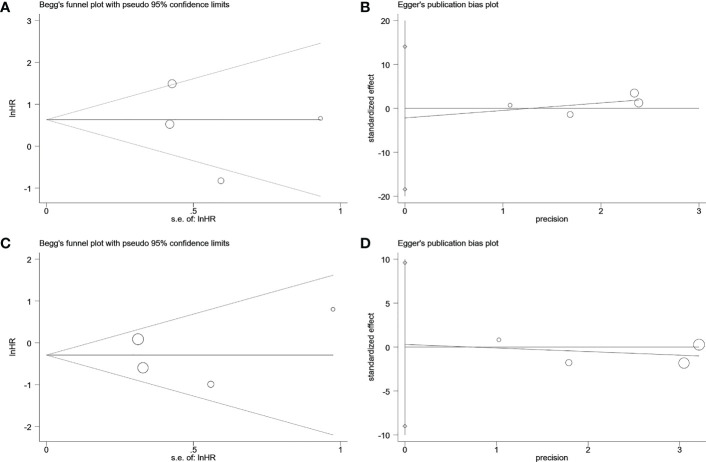
Begg’s test and egger’s test to detect publication bias. **(A)** Begg’s publication bias plot of DFS/RFS. **(B)** egger’s publication bias plot of DFS/RFS. **(C)** Begg’s publication bias plot of OS. **(D)** egger’s publication bias plot of OS. DFS, disease-free survival; RFS, relapse-free survival; OS, overall survival.

**Figure 6:**
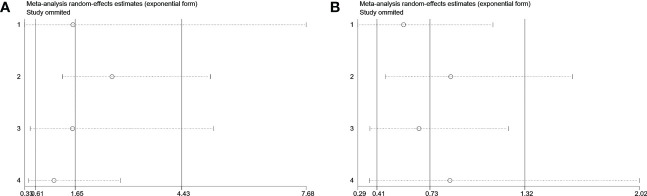
Sensitivity analyses of outcomes. **(A)** Disease-free survival. **(B)** Overall survival.

Visual inspection of the funnel plot revealed no remarkable asymmetry (**Figure 4**). Furthermore, based on Begg’s and Egger’s tests, there was no overt indication of publication bias in the included studies with respect to OS (Begg’s P = 0.734, Egger’s P = 0.903) and DFS/PFS (Begg’s P = 1.000, Egger’s P = 0.622) (**Figure 5**). A sensitivity analysis was performed by successively omitting each study. In the remaining studies, the HR for each component analysis did not exceed the predicted range. Therefore, the reliability of this meta-analysis was validated. Even when a low-quality study was omitted, the pooled findings remained consistent (**Figure 6**).

## 4 Discussion

A recent work showed that FASN overexpression was associated with poor prognosis of several types of cancer, such as ovarian, lung cancer and gastric cancer. However, a higher expression level of FASN was found to be link to poor OS, RFS, distant-metastasis free survival (DMFS) only in breast cancer patients with HER2 negative (38).

In the present study, five studies were used for the meta-analysis of 855 patients with breast cancer to examine the association of FASN expression with the clinicopathological characteristics and prognosis. Results showed that a high FASN level was link to a larger tumor size (OR, 2.04; 95% CI, 1.04-4.00; P = 0.038) and HER2 positivity (OR, 1.53; 95% CI, 1.05-2.23; P = 0.028). Higher expression of FASN had no significant effect on the prognostic indices (random-effects model pooled DFS/RFS: HR, 1.65; 95% CI, 0.61-4.43, P = 0.323; random-effects model OS: HR, 0.73; 95% CI, 0.41-1.32, P = 0.300). FASN expression was not link to the histological grade, nodal metastasis, TNM stage, Ki-67 labeling index, estrogen receptor status (ER), or progesterone receptor (PR) status.

FASN overexpression was found in many types of cancers. However, an oncogenic role of FASN has not been established. Precancerous lesions and early-stage cancer cause cell proliferation which increases the demand of oxygen and energy in these tissues. Cells in a state of hypoxia undergo anaerobic metabolism to produce energy to maintain metabolic activity. Due to the insufficiency of carbohydrates during the early stage of tumor tissue, cancer cells may activate FASN to produce long-chain fatty acids as an energy alternate for survival (39–42). In contrast to original breast cancers that had metastasized to other places, a recent study indicated that FASN was more significantly expressed in breast tumors that had spread to the brain. It was proposed that, in the brain, shortage of extracellular fatty acids may increase the need for *de novo* lipid biosynthesis and thus stimulate FASN expression under the lipid-limiting conditions (43). In addition to FASN, the expression of other genes related to fatty acid metabolic process was also found to be changed in tumor cells (38). These findings indicated the upregulation of FASN may represent as adaptation mechanism for cancer cell proliferation and survival. During the study, the results revealed a significant correlation between FASN expression level and tumor size, indicating that FASN may play an important role in tumor growth. This hypothesis was substantiated by the finding that inhibition of FASN can induce cell cycle arrest and cell apoptosis and reduce tumor size (44–47). 

Secondly, this study revealed a link between FASN overexpression and HER2 positivity. This finding could be explained by previous studies showing that FASN expression is under the control of HER2. In MDA-MB-231 cells, forced expression of HER2 increased FASN level (24). FASN expression was higher in SK-BR-3 and BT-474 cells with HER2 overexpression than in MCF-7 and MDA-MB-231 cells with low HER2 expression (48–51). Additionally, mammalian target of rapamycin (mTOR) and phosphoinositide 3-kinase (PI3K) inhibitors prevented HER2-induced FASN expression. These results suggest that HER2 regulate FASN expression through the PI3K/mTOR pathways (52).

In this study, FASN expression was not correlated with prognostic markers, such as OS or DFS, or pathological features, such as nodal metastasis, TNM stage, histological grade, ER status, PR status, and Ki-67 index, of breast cancer patients. These results partially contradict the findings of previous studies. For instance, it has been demonstrated that FASN is substantially expressed in breast cancer-associated brain metastases, which are detrimental to the prognosis (43, 53, 54). Compared with extracranial tissues, the brain has much lower lipid content in multiple complex lipid species. Ferraro et al. demonstrated that the FASN expression is higher in tumors growing in brain tissue than in extracranial tissues, with a concomitant increase in *de novo* fatty acid synthesis, while genetic and pharmacological inhibition of FASN has more inhibitory effect on breast cancer growth in the brain than in lipid-rich issues. These findings suggest that fatty acid synthesis is required for breast tumor growth in the brain. Furthermore, genetic disruption of FASN expression improved the survival of mice with breast tumors implanted in the brain (43). Therefore, FASN overexpression may contribute to the metastases of breast tumor in some specific location such as the brain, and thus have an adverse impact on the prognosis. Moreover, Jin et al. suggest that fatty acid synthesis may be an innate ability of cancer cells, and extracranially increased fatty acid synthesis may promote brain metastasis (55). These findings have important implications for the treatment of brain metastases from breast cancer as well as central nervous system malignancies.

Five studies involved in this meta-analysis, a high expression of FASN was not link to poor DFS or OS, however, subgroup analysis was not conducted in these studies (26). In another study, association between FASN expression and prognostic markers was analyzed in subgroups according to pathological subtypes, including triple-negative, HER2-overexpress, luminal A, and luminal B. A high expression of FASN was found to be link to poor RFS and DMFS only in patients with HER2-overexpress breast cancer (26).

The utilization of different metabolic pathways by various breast cancer subtypes could be a possible explanation (24). FASN expression differs significantly among subtypes with highest in HER2-overexpress breast cancers and lowest in triple-negative breast cancers. The activation of FASN-driven lipogenesis phenotype may increase the aggressiveness of HER2-overexpress breast cancer. In addition, recent studies showed that FASN may have a positive feedback effect on HER2 expression (52, 56, 57). It is possible that FASN may indirectly increase the aggressiveness of HER2-overexpress breast cancer by upregulation of HER2 expression (58).

## 5 Strengths and limitations

This study had some strengths. First, a sizable sample size was included (855 patients), resulting in increased confidence in the results. Subgroup analyses were performed to reduce heterogeneity. Second, the sensitivity analysis showed that the results were relatively consistent and did not fluctuate with the removal of studies. Furthermore, visual inspection of funnel plots and the results of Begg’s and Egger’s tests revealed that the analysis had negligible publication bias. Third, this investigation examined the relationship between breast cancer patients’ clinicopathological characteristics and FASN expression and found that FASN expression was related to tumor size and HER2 positivity.

This study had several limitations. First, the pathological classification of breast cancers was not fully indicated in the original publications, which limited the ability to perform subgroup analyses based on breast cancer subtypes. Second, FASN expression was determined by immunohistochemistry, which was subjective and did not include accurate cutoff values. Additionally, some studies have missed original data; therefore, data could only be extracted from Kaplan–Meier survival curves, which decreased the ability to accurately estimate HRs and 95% CIs. Finally, because of the lack of published studies, the data we could apply in our analysis was limited.

## 6 Conclusion

This study demonstrated that a high FASN expression was associated with tumor size and HER2 positivity, but not associated with the histological grade, tumor stage, Ki-67 labeling index, estrogen receptor (ER) status, or progesterone receptor (PR) status. These findings suggest FASN overexpression may contribute to tumor growth particularly in HER2-overexpress breast cancer. Despite that associations between FASN expression and prognostic indices were not demonstrated in this study, the possibility cannot be excluded that FASN overexpression may have adverse impacts on the prognosis on breast tumors depending on the pathological subtype and location. Future work is needed to further address this issue based on breast cancer subtype analysis. Breast cancer patients who may benefit from therapy regimens using FASN inhibitors, which may have highly toxic effects on HER2-overexpress breast cancers, could be recognized by the overexpression of HER2 of the tumor. Additionally, the results support the notion that targeted inhibition of FASN can reduce the tumor size of breast cancer patients. Its use in clinical practice to shrink tumor size, which is beneficial for reducing the surgical area and promoting postoperative incision healing is anticipated.

## Author contributions

Conception and design: BL and QP. Administrative support: RM. Provision of study materials or patients: BL and QP. Collection and assembly of data: BL, QP, and JQ. Data analysis and interpretation: BL, JQ, and Y-WW. The revision stage of the article: JZ. All authors have contributed to the manuscript and approved the submitted version. 
