# Supplementary material for: Prognostic and clinicopathological significance of fatty acid synthase in breast cancer: A systematic review and meta-analysis
Source: Front Oncol. 2023 Apr 12;13:1153076. doi: 10.3389/fonc.2023.1153076 (PMC10135304; doi:10.3389/fonc.2023.1153076)
Supplement: Supplementary file 1 [file Table_1.docx]

| **Table S1.** Details of search strategy for all databases | |
| --- | --- |
| **Database** | **Search Strategy** |
| Pubmed | ((("Breast Neoplasms"[Mesh]) OR (((((((((((((((((((((((((((((((((((((Breast Neoplasm[Title/Abstract]) OR (Neoplasm, Breast[Title/Abstract])) OR (Breast Tumors[Title/Abstract])) OR (Breast Tumor[Title/Abstract])) OR (Tumor, Breast[Title/Abstract])) OR (Tumors, Breast[Title/Abstract])) OR (Neoplasms, Breast[Title/Abstract])) OR (Breast Cancer[Title/Abstract])) OR (Cancer, Breast[Title/Abstract])) OR (Mammary Cancer[Title/Abstract])) OR (Cancer, Mammary[Title/Abstract])) OR (Cancers, Mammary[Title/Abstract])) OR (Mammary Cancers[Title/Abstract])) OR (Malignant Neoplasm of Breast[Title/Abstract])) OR (Breast Malignant Neoplasm[Title/Abstract])) OR (Breast Malignant Neoplasms[Title/Abstract])) OR (Malignant Tumor of Breast[Title/Abstract])) OR (Breast Malignant Tumor[Title/Abstract])) OR (Breast Malignant Tumors[Title/Abstract])) OR (Cancer of Breast[Title/Abstract])) OR (Cancer of the Breast[Title/Abstract])) OR (Mammary Carcinoma, Human[Title/Abstract])) OR (Carcinoma, Human Mammary[Title/Abstract])) OR (Carcinomas, Human Mammary[Title/Abstract])) OR (Human Mammary Carcinomas[Title/Abstract])) OR (Mammary Carcinomas, Human[Title/Abstract])) OR (Human Mammary Carcinoma[Title/Abstract])) OR (Mammary Neoplasms, Human[Title/Abstract])) OR (Human Mammary Neoplasm[Title/Abstract])) OR (Human Mammary Neoplasms[Title/Abstract])) OR (Neoplasm, Human Mammary[Title/Abstract])) OR (Neoplasms, Human Mammary[Title/Abstract])) OR (Mammary Neoplasm, Human[Title/Abstract])) OR (Breast Carcinoma[Title/Abstract])) OR (Breast Carcinomas[Title/Abstract])) OR (Carcinoma, Breast[Title/Abstract])) OR (Carcinomas, Breast[Title/Abstract]))) AND (("Fatty Acid Synthases"[Mesh]) OR ((((((Acid Synthases, Fatty[Title/Abstract]) OR (Fatty Acid Synthetases[Title/Abstract])) OR (Synthetases, Fatty Acid[Title/Abstract])) OR (Fatty Acid Synthase[Title/Abstract])) OR (Acid Synthase, Fatty[Title/Abstract])) OR (Synthase, Fatty Acid[Title/Abstract])))) AND (((survival) OR (mortality)) OR (prognosis)) |
| Embase | ('fatty acid synthase'/exp OR 'fatty acid synthase':ab,ti OR 'acid synthases, fatty':ab,ti OR 'fatty acid synthetases':ab,ti OR 'synthetases, fatty acid':ab,ti OR 'acid synthase, fatty':ab,ti OR 'synthase, fatty acid':ab,ti) AND ('breast neoplasm'/exp OR 'breast neoplasm' OR (('breast'/exp OR breast) AND ('neoplasm'/exp OR neoplasm)) OR 'neoplasm, breast':ab,ti OR 'breast tumors':ab,ti OR 'breast tumor':ab,ti OR 'tumor, breast':ab,ti OR 'tumors, breast':ab,ti OR 'neoplasms, breast':ab,ti OR 'breast cancer':ab,ti OR 'cancer, breast':ab,ti OR 'mammary cancer':ab,ti OR 'cancer, mammary':ab,ti OR 'cancers, mammary':ab,ti OR 'mammary cancers':ab,ti OR 'malignant neoplasm of breast':ab,ti OR 'breast malignant neoplasm':ab,ti OR 'breast malignant neoplasms':ab,ti OR 'malignant tumor of breast':ab,ti OR 'breast malignant tumor':ab,ti OR 'breast malignant tumors':ab,ti OR 'cancer of breast':ab,ti OR 'cancer of the breast':ab,ti OR 'mammary carcinoma, human':ab,ti OR 'carcinoma, human mammary':ab,ti OR 'carcinomas, human mammary':ab,ti OR 'human mammary carcinomas':ab,ti OR 'mammary carcinomas, human':ab,ti OR 'human mammary carcinoma':ab,ti OR 'mammary neoplasms, human':ab,ti OR 'human mammary neoplasm':ab,ti OR 'human mammary neoplasms':ab,ti OR 'neoplasm, human mammary':ab,ti OR 'neoplasms, human mammary':ab,ti OR 'mammary neoplasm, human':ab,ti OR 'breast carcinoma':ab,ti OR 'breast carcinomas':ab,ti OR 'carcinoma, breast':ab,ti OR 'carcinomas, breast':ab,ti) AND (survival OR prognosis OR mortality) |
| Cochrane Library | (((MeSH descriptor: [Breast Neoplasms] explode all trees)OR((Neoplasm, Breast):ti,ab,kw OR (Breast Tumors):ti,ab,kw OR (Breast Tumor):ti,ab,kw OR (Tumor, Breast):ti,ab,kw OR (Tumors, Breast):ti,ab,kw OR (Neoplasms, Breast):ti,ab,kw OR (Breast Cancer):ti,ab,kw OR (Cancer, Breast):ti,ab,kw OR (Mammary Cancer):ti,ab,kw OR (Cancer, Mammary):ti,ab,kw OR (Cancers, Mammary):ti,ab,kw OR (Mammary Cancers):ti,ab,kw OR (Malignant Neoplasm of Breast):ti,ab,kw OR (Breast Malignant Neoplasm):ti,ab,kw OR (Breast Malignant Neoplasms):ti,ab,kw OR (Malignant Tumor of Breast):ti,ab,kw OR (Breast Malignant Tumor):ti,ab,kw OR (Breast Malignant Tumors):ti,ab,kw OR (Cancer of Breast):ti,ab,kw OR (Cancer of the Breast):ti,ab,kw OR (Mammary Carcinoma, Human):ti,ab,kw OR (Carcinoma, Human Mammary):ti,ab,kw OR (Carcinomas, Human Mammary):ti,ab,kw OR (Human Mammary Carcinomas):ti,ab,kw OR (Mammary Carcinomas, Human):ti,ab,kw OR (Human Mammary Carcinoma):ti,ab,kw OR (Mammary Neoplasms, Human):ti,ab,kw OR (Human Mammary Neoplasm):ti,ab,kw OR (Human Mammary Neoplasms):ti,ab,kw OR (Neoplasm, Human Mammary):ti,ab,kw OR (Neoplasms, Human Mammary):ti,ab,kw OR (Mammary Neoplasm, Human):ti,ab,kw OR (Breast Carcinoma):ti,ab,kw OR (Breast Carcinomas):ti,ab,kw OR (Carcinoma, Breast):ti,ab,kw OR (Carcinomas, Breast):ti,ab,kw))AND ((Acid Synthases, Fatty):ti,ab,kw OR（MeSH descriptor: [Fatty Acid Synthases] explode all trees）OR（Fatty Acid Synthetases):ti,ab,kw OR (Synthetases, Fatty Acid):ti,ab,kw OR (Fatty Acid Synthase):ti,ab,kw OR (Acid Synthase, Fatty):ti,ab,kw OR (Synthase, Fatty Acid):ti,ab,kw） |
| Web Of Science | (((((((((((((((((((((((((((((((((((((TS=(Breast Neoplasm)) OR TS=(Neoplasm, Breast)) OR TS=(Breast Tumors)) OR TS=(Breast Tumor)) OR TS=(Tumor, Breast)) OR TS=(Tumors, Breast)) OR TS=(Neoplasms, Breast)) OR TS=(Breast Cancer)) OR TS=(Cancer, Breast)) OR TS=(Mammary Cancer)) OR TS=(Cancer, Mammary)) OR TS=(Cancers, Mammary)) OR TS=(Mammary Cancers)) OR TS=(Malignant Neoplasm of Breast)) OR TS=(Breast Malignant Neoplasm)) OR TS=(Breast Malignant Neoplasms)) OR TS=(Malignant Tumor of Breast)) OR TS=(Breast Malignant Tumor)) OR TS=(Breast Malignant Tumors)) OR TS=(Cancer of Breast)) OR TS=(Cancer of the Breast)) OR TS=(Mammary Carcinoma, Human)) OR TS=(Carcinoma, Human Mammary)) OR TS=(Carcinomas, Human Mammary)) OR TS=(Human Mammary Carcinomas)) OR TS=(Mammary Carcinomas, Human)) OR TS=(Human Mammary Carcinoma)) OR TS=(Mammary Neoplasms, Human)) OR TS=(Human Mammary Neoplasm)) OR TS=(Human Mammary Neoplasms)) OR TS=(Neoplasm, Human Mammary)) OR TS=(Neoplasms, Human Mammary)) OR TS=(Mammary Neoplasm, Human)) OR TS=(Breast Carcinoma)) OR TS=(Breast Carcinomas)) OR TS=(Carcinoma, Breast)) OR TS=(Carcinomas, Breast)) AND (((TS=(prognosis)) OR TS=(survival)) OR TS=(mortality)) AND ((((((TS=(Acid Synthases, Fatty)) OR TS=(Fatty Acid Synthetases)) OR TS=(Synthetases, Fatty Acid)) OR TS=(Fatty Acid Synthase)) OR TS=(Acid Synthase, Fatty)) OR TS=(Synthase, Fatty Acid)) |
