# Supplementary material for: Prognostic and clinicopathological significance of fatty acid synthase in breast cancer: A systematic review and meta-analysis
Source: Front Oncol. 2023 Apr 12;13:1153076. doi: 10.3389/fonc.2023.1153076 (PMC10135304; doi:10.3389/fonc.2023.1153076)
Supplement: Supplementary file 2 [file Table_2.docx]

**Table S2.** Detailed quality assessment of cohort studies.

| **Items of NOS** | **Included Studies** | | | | |
| --- | --- | --- | --- | --- | --- |
|  | Alo, P. L. *et al.* 1996 | Hong, Y. *et al.* 2016 | Yoshikawa, K. *et al.* 2022 | Giró-Perafita, A. *et al.* 2017 | Kim, S. *et al.*2015 |
| **Selection** |  |  |  |  |  |
| Representativeness of the exposed cohort | **★** | **★** | **★** | **★** | **★** |
| Selection of the non-exposed cohort | **★** | **★** | **★** | **★** | **★** |
| Ascertainment of exposure | **★** | **★** | **★** | **★** | **★** |
| Demonstration that outcome of interest was not present at start of study | **★** | **★** | **★** | **★** | **★** |
| **Comparability** |  |  |  |  |  |
| Comparability of cohorts on basis of the design or analysis | **★** | **★** | **★** | **★** | **★** |
| **Outcome** |  |  |  |  |  |
| Assessment of outcome |  |  |  |  |  |
| Follow-up long enough for outcomes to occur | **★** | **★** | **★** | **★** | **★** |
| Adequacy of follow up of cohorts | **★** |  | **★** |  | **★** |
| **Total** | **7** | **6** | **7** | **6** | **7** |

A study can be awarded a maximum of one star for each numbered item within the Selection and Outcome categories. A maximum of two stars can be given for Comparability. Study rates ≥6 is eligible for further analysis. NOS, Newcastle-Ottawa Scale.
