# Supplementary material for: Prognostic and clinicopathological significance of fatty acid synthase in breast cancer: A systematic review and meta-analysis
Source: Front Oncol. 2023 Apr 12;13:1153076. doi: 10.3389/fonc.2023.1153076 (PMC10135304; doi:10.3389/fonc.2023.1153076)
Supplement: Supplementary file 3 [file Table_3.docx]

| Study | Cut off value | |  |  | Grouping | |  |
| --- | --- | --- | --- | --- | --- | --- | --- |
|  | Tumor size | Ki-67 | | | TNM stage | Histological grade | |
| Alo, P. L. *et al.* 1996 | 10mm | NR | | | NR | 3 vs. 1/2 | |
| Hong, Y. *et al.* 2016 | 50mm | NR | | | Ⅲ/IV vs. Ⅰ/Ⅱ | NR | |
| Yoshikawa, K. *et al.* 2022 | 20mm | 40% | | | Ⅲ vs. Ⅰ/Ⅱ | 3 vs. 1/2 | |
| Giró-Perafita, A. *et al.* 2017 | NR | 20% | | | Ⅲ vs. Ⅰ/Ⅱ | NR | |
| Kim, S. *et al.*2015 | NR | 14% | | | NR | 3 vs. 1/2 | |

Table S3. Cut off value and grouping information of variables from included studies

NR, not reported; TNM, Tumor Node Metastasis.
